# Clinical impact of inferior mesenteric vein preservation during left hemicolectomy with low ligation of the inferior mesenteric artery for distal transverse and descending colon cancers: A comparative study based on computed tomography

**DOI:** 10.3389/fonc.2022.986516

**Published:** 2022-08-23

**Authors:** Jung Wook Suh, Jihoon Park, Jeehye Lee, In Jun Yang, Hong-Min Ahn, Heung-Kwon Oh, Duck-Woo Kim, Sung-Bum Kang

**Affiliations:** ^1^ Department of Surgery, Seoul National University Bundang Hospital, Seongnam, South Korea; ^2^ Department of Radiology, Seoul National University Bundang Hospital, Seongnam, South Korea

**Keywords:** colon cancer, left hemicolectomy, inferior mesenteric vein, low ligation, laparoscopic surgery

## Abstract

**Purpose:**

Presence of a long remnant sigmoid colon after left hemicolectomy with inferior mesenteric vein (IMV) ligation for distal transverse and descending colon cancers may be a risk factor for venous ischemia. This study aimed to evaluate the clinical impact of IMV preservation in patients who underwent left hemicolectomy with inferior mesenteric artery (IMA) preservation.

**Methods:**

We included 155 patients who underwent left hemicolectomy with IMA preservation for distal transverse and descending colon cancers from 2003 to 2020. Technical success of IMV preservation was determined by assessing pre- and post-operative patency of the IMV on computed tomography (CT) by an abdominal radiologist. Intestinal complications comprising ulceration, stricture, venous engorgement, and colitis in remnant colon were compared between the IMV preservation and ligation groups.

**Results:**

IMV was preserved in 22 (14.2%) and ligated in 133 (85.8%) patients. Surgical time, postoperative recovery outcomes, and number of harvested lymph nodes were similar in both groups. The technical success of IMV preservation was 81.8%. Intestinal complications were less common in the preservation group than in the IMV ligation group (4.5% vs. 23.3%, P=0.048). The complications in the IMV ligation group were anastomotic ulcer (n=2), anastomotic stricture (n=4), venous engorgement of the remnant distal colon (n=4), and colitis in the distal colon (n=21).

**Conclusions:**

IMV preservation may be beneficial after left hemicolectomy with IMA preservation for distal transverse and descending colon cancers. We suggest that IMV preservation might be considered when long remnant sigmoid colon is expected during left hemicolectomy with low ligation of IMA.

## Introduction

Colonic ischemia around the anastomosis site following colorectal surgery is an unusual but serious complication (1, 2). Several mechanisms underlie the development of colonic ischemia, including vascular factors such as arteriosclerosis, intestinal factors such as an increase in the intestinal pressure from constipation or other reasons, and factors associated with congestion due to poor blood circulation (3–5). A long, remnant distal stump after left hemicolectomy with low ligation of inferior mesenteric artery (IMA) and inferior mesenteric vein (IMV) ligation may cause congestive ischemic colitis due to inadequate venous circulation (3). In left hemicolectomy for left-sided colon cancer, the appropriate vascular ligation site, which includes IMV, remains controversial (6–8).

Colon cancers arising from distal transverse, splenic flexure, and proximal descending colons among left-sided colon cancers account for <10% of all colorectal cancer cases (3, 9–12), and left hemicolectomy is performed if cancer develops in that area (6–8, 13). The left colic artery is ligated to retrieve the nodes in this area and the left branch of the middle colic artery is ligated to harvest the surrounding nodes (14–18). IMV is usually ligated at the inferior margin of the pancreas to ensure sufficient lymph node harvest (3, 19, 20), although studies have not yet been conducted regarding the appropriate ligation site for the IMV. IMV ligation near the root may lead to an imbalance in the venous return when the preserved IMA maintains sufficient blood supply. Four cases of congestive ischemic colitis due to venous return imbalance were reported (3). However, to date, no studies have been conducted to evaluate congestive ischemic colitis, occurring in long remnant sigmoid colon after left hemicolectomy with low ligation of IMA, and the clinical benefits of IMV preservation.

The aim of this study was to compare the clinical outcomes of IMV preservation with those of IMV ligation in laparoscopic left hemicolectomy with low IMA ligation for distal transverse and descending colon cancers and evaluate the technical success rate of IMV preservation through CT.

## Materials and methods

### Study population

Patients who underwent curative laparoscopic left hemicolectomy for distal transverse and descending colon cancers at Seoul National Bundang Hospital from March 2003 to January 2020 were included in this study, and their electronic medical records were retrospectively reviewed. Left hemicolectomy was performed for cancers arising from the distal transverse, splenic flexure, and proximal descending colons. Until December 2018, IMA low ligation (IMA preservation and left colic artery ligation) and IMV ligation were performed along with left hemicolectomy. From December 28, 2018, IMV preservation was selectively performed in patients who were expected to have long remnant sigmoid colons after left hemicolectomy with low ligation of IMA. Patients with conversion to laparotomy or resection margin positive or palliative surgery were excluded. Patient characteristics were registered as follows: age, sex, American Society of Anesthesiologists (ASA) score, white blood cell count (WBC), serum albumin level, serum creatinine level, clinical T classification, and clinical N classification. Tumor stage was assessed according to the Union for International Cancer Control TNM staging (8th edition). This study was approved by the institutional review board of the Seoul National Bundang Hospital (IRB No. B-2108/702-106), and the need for informed consent was waived because of the retrospective nature of the study.

### Surgical techniques

**Figure 1:**
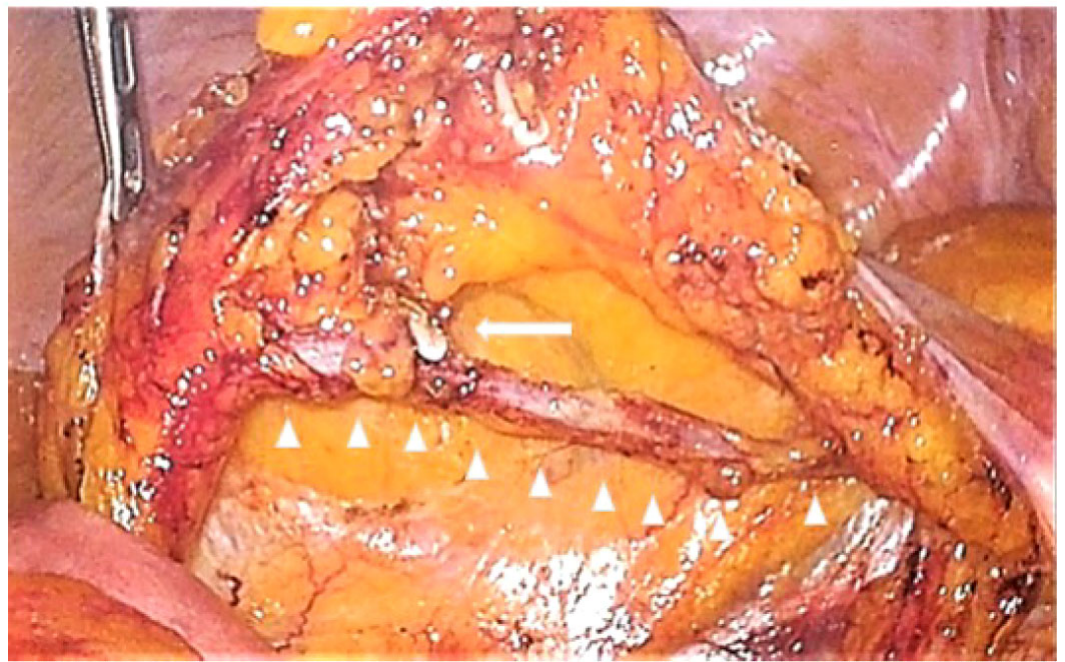
Preserved inferior mesenteric vein after dissection of lymph nodes around the inferior mesenteric vein. The left side of the figure is the cranial side, and the right side is the caudal side. The triangles indicate the skeletonized inferior mesenteric vein, and the arrow indicates the ligation of the left colic vein.

Laparoscopic surgery was performed at tertiary referral hospitals by surgeons who performed more than 200 laparoscopic colorectal surgeries annually. Access for laparoscopic surgery was obtained using five trocars. Endohooks, monopolar electrosurgical devices with hook-shaped tips (A6282, Olympus Medical Systems Corp, Tokyo, Japan) were used for dissection. Lymph node dissection was performed from the IMA origin through the medial to lateral approach. After examining the site where the left colic artery branches were, the left colic artery was ligated using clips. Dissection of the lymph node surrounding the IMV was initiated from the IMV around the ligated left colic artery. IMV skeletonization was performed through sharp dissection until the lower border of the pancreas (**Figure 1**). During this process, two or three veins joining the IMV could be observed. After the left branches of the middle colic vessels were ligated, the splenic flexure and transverse colon were freed from the greater omentum. The splenocolic ligament was then divided, and the left colon was retracted toward the midline; subsequently, the colon was freed from the left lateral abdominal wall by sharp dissection. The left ureter and gonadal vessel were identified and saved. After full mobilization of the left colon, an extracorporeal double-stapled colo-colonic anastomosis was performed between the distal transverse and sigmoid colon. In case of IMV ligation, the procedures were the same as above, except that the IMV was ligated and divided below the pancreatic margin.

### Outcomes

Perioperative and postoperative outcomes (surgical time, estimated blood loss, blood transfusion, first passage of flatus, and hospital stay), pathologic features (number of harvested lymph nodes, tumor differentiation, pathologic T and N classification, lymphatic invasion, venous invasion, and perineural invasion) and morbidities over Clavein–Dindo class II were also compared between IMV preservation and ligation.

**Figure 2:**
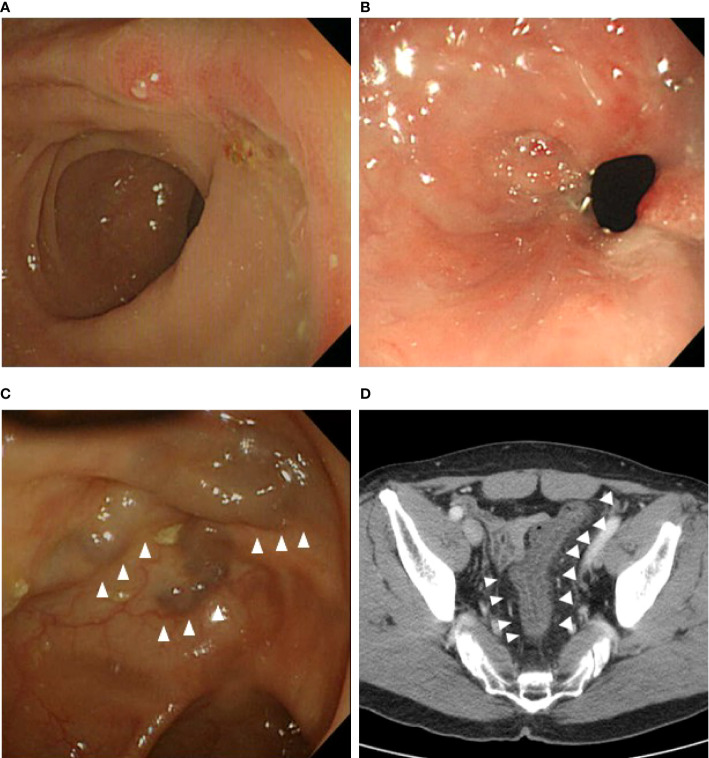
Intestinal complications on computed tomography or colonoscopy during follow-up. **(A)** Anastomotic ulcer. **(B)** Anastomotic stricture. **(C)** Venous engorgement at the remnant distal bowel. **(D)** Colitis with thickened colon wall at the remnant distal bowel.

We investigated the postoperative intestinal complications, and the proportion of patients with postoperative complications was compared between those who underwent IMV preservation and those who underwent ligation. We defined intestinal complications as ulceration, stricture, venous engorgement, and colitis in the remnant colon and confirmed these using computed tomography (CT) or colonoscopy. Previous diagnoses of inflammatory bowel disease with involvement of other intestinal sites were not counted. CT and endoscopic findings were confirmed by reviewing the medical records of those who underwent regular postoperative examinations. An anastomotic ulcer was defined as mucosal breakdown localized solely to the anastomotic site observed *via* colonoscopy (**Figure 2A**) (21). Anastomotic strictures were present if (1) significant force was required to pass the colonoscope beyond the anastomosis and this produced visible trauma after passing through, and/or (2) balloon dilation was required for passage of the colonoscope to visualize the remainder of the colon (**Figure 2B**) (21). Venous engorgement of the remnant distal stump was defined as prominent enlarged veins and varices on CT or colonoscopy (**Figure 2C**) (21). Colitis of the remnant distal stump, caused by inadequate venous circulation, ranging from mild mesenteric panniculitis to severe congestive ischemic colitis requiring surgery, was diagnosed using CT and colonoscopy (**Figure 2D**) (3, 22–25).

**Figure 3:**
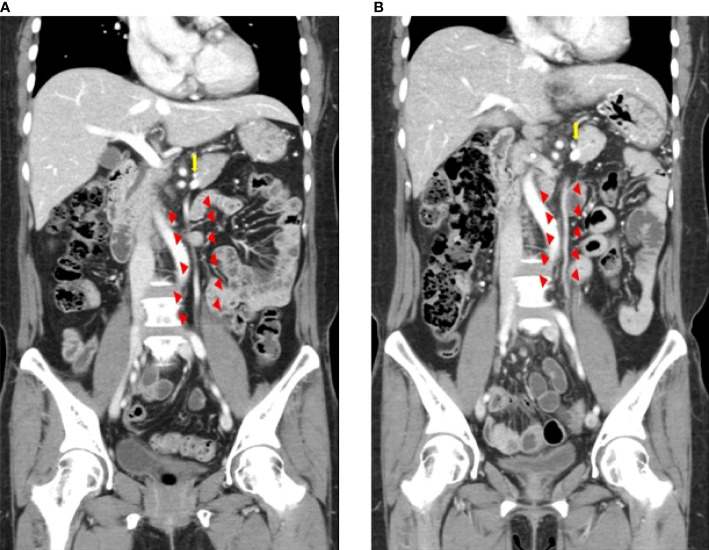
Inferior mesenteric vein preservation confirmed on computed tomography images before **(A)** and after surgery **(B)** Triangles indicate the inferior mesenteric vein. The arrow indicates the splenic vein.

The technical success rate was measured among patients who underwent IMV preservation. The technical success of IMV preservation was determined by assessing the post-operative patency of IMV on CT. A board-certified abdominal radiologist (J.H.P, 11 years of clinical experience) retrospectively reviewed the pre- and post-operative CT images and rated the patency of the IMV using a 3-point scale, where grade 1 indicated no change in patency, grade 2 indicated that the patency had decreased by 30% or more, and grade 3 indicated that the IMV was not visualized (**Figure 3**) (26). The radiologist was aware of patient eligibility criteria and might have determined the surgical technique through the image review, but was blinded to the purpose of this study and other clinical information.

All patients underwent one typical follow-up schedule according to our standard postoperative surveillance protocol for colorectal cancer (27, 28). Postoperatively, outpatient visits, physical examinations, and tests for serum carcinogenic antigen (CEA) levels were performed every 3 months for 2 years and every 6 months for 5–8 years thereafter. Follow-up CT of the chest, abdomen, and pelvis was performed every 6 months during the first 2 years and then yearly for the next 3 years. Endoscopic assessment was performed 1 year after the operation and every 2 years thereafter. Positron emission tomography was performed if required, when recurrence was suspected.

### Statistical analysis

Continuous variables are presented as their means with standard deviation or medians with interquartile ranges for normally distributed or non-normally distributed variables, and categorical variables are presented as counts with percentages. All tests were two-sided, and differences were considered significant at p-values of < 0.05. Continuous data were compared using Student’s t-test or the Mann–Whitney U test for normally or non-normally distributed variables, respectively. Chi-square and Fisher’s exact tests were used, when appropriate, to compare categorical variables. Statistical analyses were performed using the Statistical Package for Social Sciences (SPSS) version 21 (IBM Corp., Armonk, NY., USA).

## Results

A total of 155 patients underwent laparoscopic left hemicolectomy with low ligation of IMA, with 22 (14.2%) undergoing IMV preservation (IMV preservation group) and 133 (85.8%) undergoing IMV ligation (IMV ligation group). **Table 1** reports the patients’ baseline characteristics. The median age was 64 years, and 97 patients (62.6%) were men. None of the preoperative variables (age, sex, body mass index, ASA grade, WBC count, albumin, creatinine, clinical T classification, and clinical N classification) were significantly different between the two groups.

**Table 1:** Patients’ baseline characteristics.

Variable	IMV preservation (n = 22)	IMV ligation (n = 133)	*P*-value
Age, median (IQR), years	63.5 (57–72)	64.0 (56–72)	0.947
Sex, n (%)			0.912
Male	14 (63.6)	83 (62.4)	
Female	8 (36.4)	50 (37.6)	
Body mass index, median (IQR), kg/m^2^	24.1 (22–26)	24.4 (22–26)	0.776
ASA grade, n (%)			0.174
I, II	17 (77.3%)	117 (88.0)	
III, IV	5 (22.7%)	16 (12.0)	
WBC count, median (IQR), cells/mm^3^	7.3 (6–8)	6.1 (5–7)	0.053
Albumin, median (IQR), g/dL	4.2 (4–5)	4.2 (4–5)	0.555
Creatinine, median (IQR), mg/dL	0.8 (0.7–0.9)	0.9 (0.7–1)	0.054
cT classification, n (%)			0.741
cT0	6 (27.3)	28 (21.1)	
cT1	3 (13.6)	11 (8.3)	
cT2	1 (4.5)	14 (10.5)	
cT3	9 (40.9)	65 (48.9)	
cT4	3 (13.6)	15 (11.3)	
cN classification, n (%)			0.601
cN0	15 (68.2)	89 (66.9)	
cN+	7 (31.8)	44 (33.1)	

Data are presented as n (%) or median (interquartile range, IQR).

IMV, inferior mesenteric vein; ASA, American Society of Anesthesiologists; WBC, white blood cell; c, clinical stage.

In terms of perioperative outcomes, duration of surgery was similar in both groups (**Table 2**). No significant between-group differences were observed in terms of estimated blood loss and blood transfusion. Postoperative outcomes (first passage of flatus and hospital stay) were also similar between the two groups. The median number of harvested lymph nodes was 36.5 in the IMV preservation group and 37 in the IMV ligation group, but there was no significant difference between two groups. No significant differences were found between the two groups in terms of tumor differentiation and pathologic T or N classification and lymphatic, venous, and perineural invasion.

**Table 2:** Peri- and post-operative outcomes and pathologic features.

Variable	IMV preservation (n = 22)	IMV ligation (n = 133)	*P*-value
Duration of surgery, median (IQR), min	200 (168–214)	185 (155–235)	0.383
EBL, median (IQR), mL	30 (30–45)	100 (30–100)	0.508
Blood transfusion, n (%)			1.000
No	0 (0)	2 (1.5)	
Yes	22 (100)	131 (98.5)	
First passage of flatus, median (IQR), days	3 (2–4)	3 (2–4)	0.864
Hospital stay, median (IQR), days	6 (5–8)	7 (6–8)	0.669
Number of harvested lymph nodes, median (IQR)	36.5 (27–44)	37 (27–48)	0.937
Tumor differentiation, n (%)			0.218
Well/moderate	22 (100)	121 (91.0)	
Poor/others	0 (0)	12 (9.0)	
pT classification, n (%)			0.267
pT0	0 (0)	7 (5.3)	
pT1	5 (22.7)	32 (24.1)	
pT2	3 (13.6)	8 (6.0)	
pT3	9 (40.9)	71 (53.4)	
pT4	5 (22.7)	15 (11.3)	
pN classification, n (%)			0.995
pN0	14 (63.6)	84 (63.2)	
pN1	6 (27.3)	36 (27.1)	
pN2	2 (9.1)	13 (9.8)	
Lymphatic invasion (+), n (%)	10 (45.5)	36 (27.1)	0.080
Venous invasion (+), n (%)	5 (22.7)	22 (16.5)	0.479
Perineural invasion (+), n (%)	10 (45.5)	44 (33.1)	0.259

Data are presented as n (%) or median (interquartile range, IQR).

Abbreviations: IMV, inferior mesenteric vein; EBL, estimated blood loss; p, pathologic stage.

The technical success rate was 81.8% (18/22) in the IMV preservation group. Fifteen patients (68.2%) had grade 1 patency, three (13.6%) had grade 2 patency, and four patients (18.2%) had grade 3 patency.

Intestinal complications were significantly less common in the IMV preservation group than in the IMV ligation group (1 patient (4.5%) vs. 31 patients (23.3%), P=0.048). The intestinal complications of the IMV ligation group were anastomotic ulcer (n=2), anastomotic stricture (n=4), venous engorgement of the remnant distal colon (n=4), and colitis of the distal colon (n=21). In contrast, in the IMV preservation group, one patient (4.5%) had colitis that occurred in the remnant colon (**Table 3**). Regarding morbidity (**Table 4**), 2 patients had a Clavien–Dindo classification of III or higher. One was a 67-year-old female patient who underwent anterior resection of the remnant distal stump due to ischemic colitis that occurred 13 months after surgery. The other was a 48-year-old male patient who underwent colonoscopic bougination for an anastomotic stricture that occurred 3 months after surgery. Two patients and one patient were hospitalized for colitis and stricture, respectively, and conservative treatment was administered. All these complications developed in the patients in the IMV ligation group.

**Table 3:** Intestinal complications on computed tomography and using colonoscopy.

	IMV preservation (n = 22)	IMV ligation (n = 133)	*P*-value
Total, n (%)	1 (4.5)	31 (23.3)	0.048
Ulcer	0 (0)	2 (1.5)	
Stricture	0 (0)	4 (3.0)	
Venous engorgement	0 (0)	4 (3.0)	
Colitis	1 (4.5)	21 (15.8)	

Data are presented as n (%).

IMV, inferior mesenteric vein.

**Table 4:** Morbidities in the inferior mesenteric vein ligation group.

No.	Sex/age	Location	Findings	Tools of diagnosis	Management	Clavien–Dindo classification
1	F/67	Distal transverse colon	Ischemic colitis	CT/Colonoscopy	Anterior resection	IIIb
2	M/58	Splenic flexure colon	Colitis	CT	Conservative treatment	II
3	M/58	Descending colon	Stricture	CT	Conservative treatment	II
4	M/75	Descending colon	Colitis	CT	Conservative treatment	II
5	M/48	Descending colon	Stricture	Colonoscopy	Colonoscopic bougination	IIIa

No., Number; F, female; M, male; CT, computed tomography; IMV, inferior mesenteric vein.

The median follow-up duration was 32.0 (IQR 25.0–36.0) months in 22 patients in the IMV preservation group. Lung metastasis was detected 27 months after surgery in one patient (4.5%), and metastasectomy was performed. In addition, there was no recurrence or death in the other patients.

## Discussion

This study showed that IMV preservation may have clinical benefits in laparoscopic left hemicolectomy with low ligation of the IMA. Congestive ischemic colitis did not occur in the IMV preservation group, and the occurrence of intestinal complications was lower than in the IMV preservation group. These results provide objective evidence that IMV preservation is considered in cases of a remnant long sigmoid colon after left hemicolectomy with low ligation of the IMA.

Congestive ischemic colitis may occur if systemic circulation through the rectal vein is insufficient to compensate for the lack of drainage through the IMV during IMV ligation in left hemicolectomy with low IMA ligation. Accordingly, we have preserved the IMVs of patients who had been expected to have a long remnant sigmoid colon after left hemicolectomy with low ligation of IMA since December 28, 2018. Fuji et al. published a case report describing congestive ischemic colitis that occurred in four (2.1%) of 191 patients who underwent left-sided colectomy in which the arterial branch (superior rectal artery) was preserved and the IMV was cut near the root (3). In our study, three patients (2.25%) with colitis required treatment, and one required surgical treatment. This incidence is similar to that in previous case reports on congestive ischemic colitis in which a similar or higher incidence (0.45%–24%) of colonic ischemia was reported following IMA root ligation during colorectal cancer surgery (1, 4, 13, 29–34). Symptoms occurring at the anastomosis site due to inadequate venous drainage are similar to those related to IMV occlusion due to protein S or C deficiency or antiphospholipid antibody syndrome, such as mesenteric panniculitis of the colon or colitis (22–25). Anastomotic ulcer and stricture are caused by repeated anastomotic inflammation, and venous engorgement of the remnant distal colon indicates imbalances in venous return (3, 21–25). In our study, the extent of colitis caused by inadequate venous circulation ranged from mild mesenteric panniculitis to severe congestive ischemic colitis requiring surgery.

One of the concerns about IMV preservation was oncologic safety. IMV ligation was performed to remove the lymph nodes around the IMV (22–25). During IMV preservation, the surrounding lymph nodes could be sufficiently removed through IMV skeletonization. In our study, the number of harvested lymph nodes did not differ significantly between the two groups. Hence, lymph node removal was adequate in the IMV preservation group compared with the IMV ligation group. In a retrospective study with a median follow-up of 46 months, local recurrence was found in 4% and distant recurrence in 16% of patients undergoing left hemicolectomy (7). The 1-, 2-, and 5-year overall survival rates after left hemicolectomy, which were based on a large multicenter European sample of patients with splenic flexure colon cancer, were 97.8%, 95.2%, and 76.3% and disease-free survival rates were 86.2%, 78.8%, and 70.3%, respectively (8). In our study, the overall survival rate at 32 months of median follow-up was 100%. In addition, the disease-free survival rate was 95.5%, which was limited by the small sample size and short follow-up period. However, our results were not inferior to those of previous studies. Another concern is that technical difficulties could lengthen the duration of surgery and increase the amount of bleeding during surgery. In our study, the duration of surgery in the IMV preservation group did not differ from that in the IMV ligation group. We assume that certain devices, such as advanced laparoscopic cameras and endohook-type monopolar electrodes, help overcome technical difficulties associated with IMV preservation. With the development of laparoscopic camera equipment blood vessels can be observed more precisely and be enlarged during surgery (35). The three-dimensional scope provides depth perception in the operating field. In addition, the use of endohook-type monopolar electrodes during laparoscopic surgery may provide more meticulous lymph node dissection and lower morbidity (36).

In our study, the patency of the preserved IMV was visualized in 81.8% of patients, which was higher than the 41% reported in the study by Yoon et al. in which patency was evaluated after the preservation of splenic veins (37). We suggest that IMV preservation may have clinical benefits in laparoscopic left hemicolectomy with ligation of the left colic artery, even though patency may not be maintained, since, compared to arteries, veins have fewer muscle and elastic fibers and transport blood under lower pressure and at a lower velocity (37).

This study had some limitations. First, it was a single-center retrospective study, and selection biases may have been unavoidable. However, the baseline characteristics of the study patients did not differ between the two groups, indicating that the two groups were somewhat evenly distributed. Second, the number of reoperations due to congestive ischemic colitis occurring in the IMV ligation group was few and there were no major complications in the IMV preservation group; thus, it was difficult to make an accurate comparison between the two groups. Therefore, we compared the clinical outcomes caused by venous return dysfunction on CT or colonoscopy from anastomosis to the remnant distal colon after left hemicolectomy with low ligation of IMA. Finally, since the IMV preservation approach was introduced in 2018, the follow-up period was short. Therefore, we are unable to comment on the long-term outcomes of IMV preservation in patients with distal transverse and descending colon cancers. However, the number of harvested lymph nodes was not significantly different between the groups. Therefore, further large-scale randomized controlled studies are required to overcome the above limitations and confirm the clinical benefits of IMV preservation in laparoscopic left hemicolectomy.

This is the first CT-based study showing that IMV preservation may be beneficial after laparoscopic left hemicolectomy with low ligation of IMA for distal transverse and descending colon cancers. IMV preservation is technically feasible and can be performed without substantially increasing the duration of surgery. Therefore, IMV preservation is considered when a long remnant sigmoid colon is expected after left hemicolectomy with low IMA ligation.

## Data availability statement

The raw data supporting the conclusions of this article will be made available by the authors, without undue reservation.

## Ethics statement

This study was reviewed and approved by institutional review board of the Seoul National Bundang Hospital (IRB No. B-2108/702-106). Written informed consent for participation was not required for this study in accordance with the national legislation and the institutional requirements.

## Author contributions

Jung Wook Suh and Jihoon Park contributed equally to this work. All authors contributed to the article and approved the submitted version.

## Acknowledgments

We would like to thank Editage (www.editage.co.kr) for English language editing.

## Conflict of interest

The authors declare that the research was conducted in the absence of any commercial or financial relationships that could be construed as a potential conflict of interest.

## Publisher’s note

All claims expressed in this article are solely those of the authors and do not necessarily represent those of their affiliated organizations, or those of the publisher, the editors and the reviewers. Any product that may be evaluated in this article, or claim that may be made by its manufacturer, is not guaranteed or endorsed by the publisher.
